# 3D reconstruction of the cerebellar germinal layer reveals tunneling connections between developing granule cells

**DOI:** 10.1126/sciadv.adf3471

**Published:** 2023-04-05

**Authors:** Diégo Cordero Cervantes, Harshavardhan Khare, Alyssa Michelle Wilson, Nathaly Dongo Mendoza, Orfane Coulon-Mahdi, Jeff William Lichtman, Chiara Zurzolo

**Affiliations:** ^1^ Membrane Traffic and Pathogenesis, Institut Pasteur, Université Paris Cité, CNRS UMR 3691, F-75015 Paris, France.; ^2^ Université Paris-Saclay, 91405 Orsay, France.; ^3^ Department of Neurology, Department of Psychiatry, Icahn School of Medicine at Mount Sinai, New York, NY 10029, USA.; ^4^ Research Center in Bioengineering, Universidad de Ingeniería y Tecnología-UTEC, Lima 15049, Peru.; ^5^ Department of Molecular and Cellular Biology, Center for Brain Science, Harvard University, Cambridge, MA 02138, USA.

## Abstract

The difficulty of retrieving high-resolution, in vivo evidence of the proliferative and migratory processes occurring in neural germinal zones has limited our understanding of neurodevelopmental mechanisms. Here, we used a connectomic approach using a high-resolution, serial-sectioning scanning electron microscopy volume to investigate the laminar cytoarchitecture of the transient external granular layer (EGL) of the developing cerebellum, where granule cells coordinate a series of mitotic and migratory events. By integrating image segmentation, three-dimensional reconstruction, and deep-learning approaches, we found and characterized anatomically complex intercellular connections bridging pairs of cerebellar granule cells throughout the EGL. Connected cells were either mitotic, migratory, or transitioning between these two cell stages, displaying a chronological continuum of proliferative and migratory events never previously observed in vivo at this resolution. This unprecedented ultrastructural characterization poses intriguing hypotheses about intercellular connectivity between developing progenitors and its possible role in the development of the central nervous system.

## Teaser

A connectomic-based approach reveals nano-scale tubes connecting cerebellar granule cells during postnatal development in mice.

## INTRODUCTION

The principles that regulate the development of brain connectivity and modulation of synaptic transmission are just beginning to be understood (*1*, *2*). From the historical controversy between the reticulon and neuron theories (*3*, *4*), it is plausible that synaptic transmission does not solely explain how brain cells communicate. During development, brain cells communicate to orchestrate cell divisions and migrations in the absence of synapses; this intercellular communication relies on gap junctions and mitogens (*5*), which contribute to the establishment of neuronal assemblies.

Another mechanism of intercellular communication allowing the exchange of molecules and entire organelles between connected cells is stable intercellular bridges (IBs) (*6*), which arise from incomplete cytokinesis, part of the cell division process during which the cytoplasm of a single cell is stalled as it divides into two daughter cells. Unlike cytokinetic bridges (CBs) that form and are normally cleaved in a series of well-known events during cytokinesis (*7*), the mechanisms regulating the development of IBs and their function are not yet understood. Today, IBs are considered an evolutionarily conserved process across species (from insects to humans) that occur in female and male germ lines; they have rarely been observed in somatic tissues of invertebrates and never previously in the brain (*6*).

The cerebellum, a brain region vital for balance control and motor movements, arises from a series of finely orchestrated cellular division and migratory events. These processes are paramount for proper development of the cerebellar cortex and in particular, the postnatal development of granule cell (GC) progenitors within the transient, germinal, external granular layer (EGL) (*8*).

**Fig. 1.**
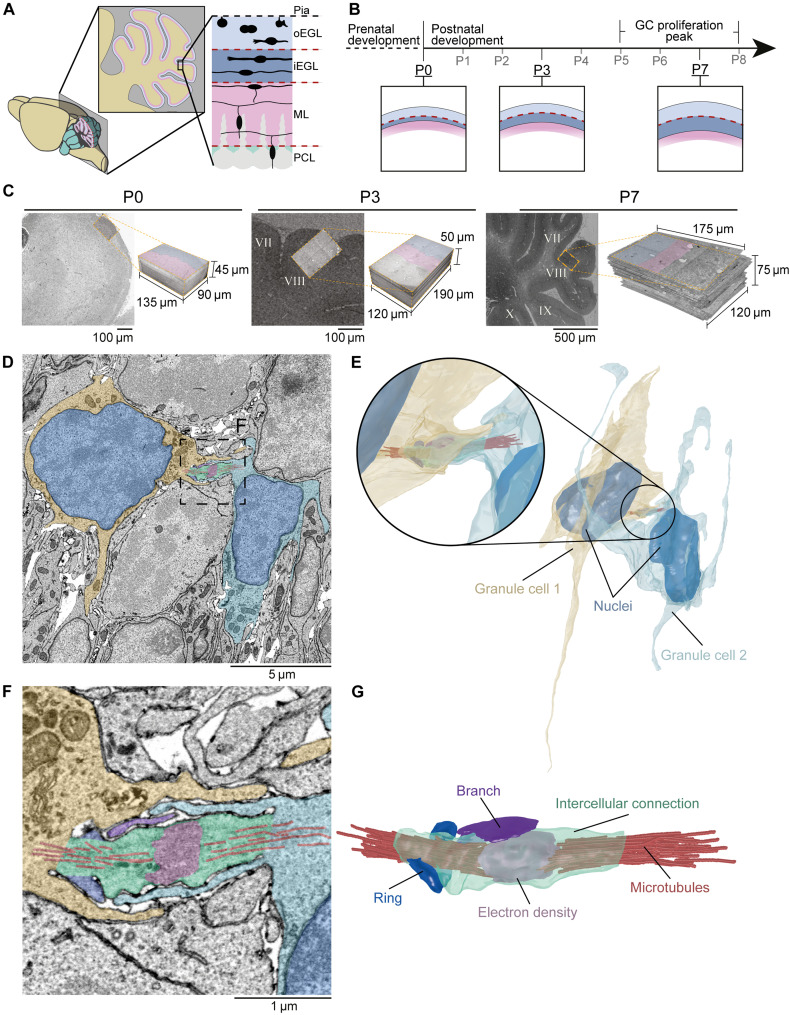
Identification of cerebellar GC intercellular connectivity. (**A**) Schematic representation of a mouse cerebellar cortex sagittal cross section. Sagittal view of the three outer laminae of the cerebellar cortex: the EGL (blue) divided into outer and inner EGL (oEGL and iEGL), the molecular layer (ML; pink), and Purkinje cell layer (PCL; green). (**B**) During pre- and postnatal development, GCs populate the oEGL via mitosis, resulting in a sequential expansion of the EGL (P0, P3, and P7 inserts) and a proliferation peak between postnatal days 5 and 8 (P5 and P8). Postmitotic GCs arising from these divisions then migrate tangentially in the iEGL. To become mature neurons, postmitotic GCs migrate radially through the ML and PCL and settle in the internal granular layer (IGL), where they establish synapses with Golgi cells and Mossy fibers (not shown). (**C**) P0, P3, and P7 electron micrographs and 3D volumes prepared using serial-sectioning scanning electron microscopy (ssSEM); lobule VIII for P3 and P7 volumes. (**D**) 2D electron micrograph showing GCs (yellow and blue) in the EGL of the P7 volume bridged by an intercellular connection (IC; green). (**E**) 3D reconstructions of D. (**F** and **G**) Zoomed-in micrograph (F) and 3D reconstruction (G) of (D) and (E), respectively, showing microtubules (red) that emanate into both GCs, an electron-dense region at the center of the IC (pink), an IC branch (magenta), and an incomplete ring (blue) extruding from the IC.

GCs are thought to transition through symmetric and asymmetric divisions exclusively in the outer-EGL (oEGL) sublayer before gradually exiting the cell cycle and differentiating into migratory, postmitotic GC neurons in the inner-EGL (iEGL) (Fig. 1A) (*9*). The ratio of proliferative to migratory GCs in the oEGL and iEGL is not consistent throughout the lifetime of the EGL; the EGL is mostly composed of dividing cells in early postnatal stages and migratory cells in later ones (*10*). The expansion of the EGL reaches a peak in proliferation between postnatal days 5 and 8 (P0 and P8; in mice) (*11*) (Fig. 1B), during which GCs born in earlier days can be found migrating in the iEGL. Although previous studies have focused on the molecular pathways regulating the exit of GCs from the EGL (*12*), the lack of a morphological survey of GCs at high resolution has hindered a detailed description of the postnatal, developing cerebellar cortex.

To investigate the three-dimensional (3D) ultrastructure of GCs in the cerebellar cortex during early stages of postnatal development at high resolution, we chose to use a serial-sectioning scanning electron microscopy (ssSEM)–based connectomic approach, a state-of-the-art technology that was developed to study brain connectivity at nanometer resolution (*13*, *14*). Despite great technological advancements in the connectomics field, producing high-resolution 3D ssSEM volumes is a difficult and time-consuming process (*13*, *15*, *16*). Therefore, insights obtained into this methodology have typically been limited to one or few volumes/animals. To explore our questions, we prepared one volume of a 0-day-old mouse (P0) cerebellum and reused two previously published volumes obtained from the cerebella of mice at P3 and P7 (*14*).

An assessment of GCs in the EGL of these volumes led to the discovery of GCs connected by membrane-bound cytoplasmic, intercellular connections (ICs) throughout the depth of the EGL. Although some of these connections exhibited similarities with (CBs), most displayed features that were never previously described and which could be unique to the brain.

In summary, our systematic examination of the neural germinal layer of the cerebellar cortex by ssSEM revealed frequent cytoplasmic interconnections between developing neural cells. Given that persistent CBs, IBs, have never been observed in the brain, our work highlights unknown morphological and cytological features of developing GCs in the mouse cerebellum and provides a perspective on the mechanisms regulating brain development as a whole.

## RESULTS

### Cerebellar GCs linked by ICs

To gain insight into the cerebellar cortex and the ulrastructural properties of GCs in the EGL, we studied three large-scale ssSEM volumes obtained from mice pups at P0, P3 (*14*), and P7 (*14*) spanning 5.5 × 10^5^ μm^3^, 1.1 × 10^6^ μm^3^, and 1.7 × 10^6^ μm^3^, respectively (Fig. 1C). To analyze the morphological features of GCs in the EGL, we manually segmented the soma and protrusions of cells in all three volumes using the manual ssSEM volume annotator, Volume Annotation and Segmentation Tool (VAST) (movie S1) (*17*).

During segmentation of a few cells in each volume, we found pairs of GCs linked by a membranous IC resembling a CB that forms toward the end of mitosis (Fig. 1, D and E, and fig. S1). CBs are microtubule-rich connections that play a vital role in the abscission of dividing cells; however, observations of CBs have been limited to in vitro models (*18*, *19*). Intracellular segmentation and 3D reconstruction of an IC in the EGL at P7 revealed two features previously ascribed to CBs: (i) an electron-dense structure within the tube, which could indicate the presence of a midbody (MB); and (ii) microtubules emanating from the dense region into both cells (Fig. 1, F and G, and movie S2) (*20*).

To the best of our knowledge, IC-like structures formed between GCs in the EGL were never reported. Thus, to quantify and characterize this phenomenon, we decided to execute a systematic screen of all ICs in the EGL. Performing an in-depth analysis that would enable us to understand the localization, morphology, and subcellular architecture of connected GCs would require enormous amounts of time and human resources, since manually screening thousands of cells across all volumes (up to 2000 cells per volume) would require numerous years and personnel to complete (*21*, *22*). Therefore, we decided to focus on the characterization of only one volume. Among the three volumes we had available at our disposable, we chose P7 to capture a relatively large number of proliferative and migratory GCs resulting from a peak in proliferation between P5 and P8 (*11*, *23*).

**Fig. 2.**
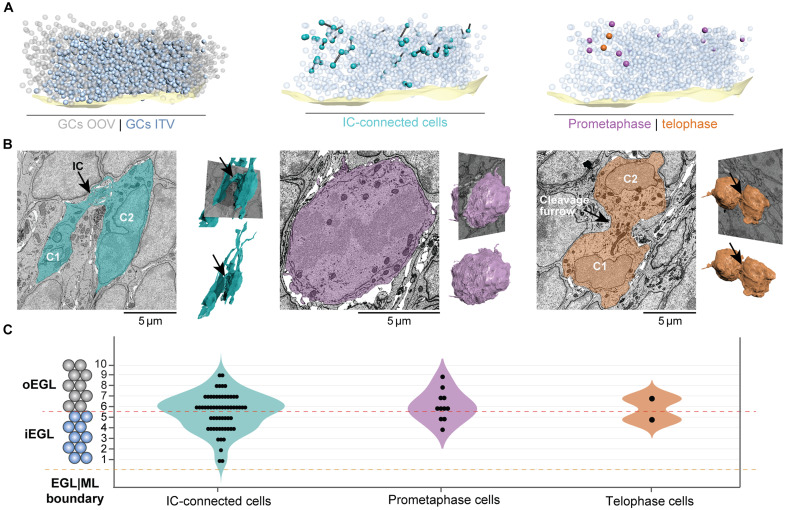
Characterization of GCs in the ssSEM volume. (**A**) Left: 3D map of EGL/ML boundary (light yellow) and 2042 GCs in the EGL of the volume: 1110 partially outside of the volume (OOV; gray) and 932 inside the volume (ITV; blue); middle: 3D map of IC-connected GCs (cells, cyan; ICs, black cylinders); right: 11 GCs in prometaphase (purple) and 2 in telophase (orange) distributed ITV. (**B**) 2D cross sections along the sagittal plane of the EGL and 3D reconstructions of GCs in different cell cycle stages. Left: IC-connected GCs (black arrow) with well-formed nuclei and long protrusions; middle: GC in prometaphase exhibiting a broken nuclear envelope and a round shape without protrusions; right: GCs in telophase showing fully formed nuclei, without protrusions, and connected by a cleavage furrow (black arrow). (**C**) Distribution of GCs interconnected by ICs (*n* = 60), prometaphase (*n* = 11), and telophase (*n* = 2) throughout the EGL shows that all events take place in the inner (layers 1 to 5) and outer (layers 6 to 10) EGL.

To narrow down our region of interest, GCs of the EGL and not cells migrating radially through the molecular layer (ML) (Fig. 1A) (the neighboring region that also contains other interneurons), we first determined the EGL/ML boundary through an automatic and reproducible approach. This approach was developed by us using a 2D convolutional neural network (CNN) architecture that was iteratively applied to the slices of our volume, which helped us to markedly reduce the manual effort and time required to distinguish the two layers (see Supplementary Methods, Fig. 2A, and S2).

### GCs carry out mitotic divisions across the EGL

We manually identified and counted all GCs in the P7 volume (see Supplementary Methods) and identified a total of 2042 GCs. Of these, 1110 cell somas were partially outside the volume (OOV) as they were located at the edges of the volume. We focused our study on the 932 cells with nuclei inside the volume (ITV) (Fig. 2A).

A subcellular diagnosis of every ssSEM slice occupied by each GC revealed 60 of 932 GCs forming ICs (6.4% of cells, a ratio of 1:15.5 connected-to-nonconnected cells) (Fig. 2, A and B). In addition, we also identified 11 of 932 GCs in prometaphase (1.2% of cells), evidenced by a disassembled nuclear envelope (*24*) and 2 of 932 GCs in telophase (0.2% of cells), identified on the basis of their spherical-shaped morphology, assembled nuclei, and wide, cleavage furrow (Fig. 2, A and B).

This observation prompted us to investigate whether the laminar location of these three distinct cellular events was consistent with previous reports of GCs exclusively dividing in the EGL, as well as to test the proximity of ICs in relation to dividing GCs. To this end, we split the EGL into two constituent, five-cell-deep sublayers representing the oEGL and iEGL, as previously reported in the literature (*25*) (Fig. 2C). Layer numbers were manually assigned to every GC by counting the number of cells between the ML and the GC of interest. Using this definition, 25 (42%) of IC-connected GCs belonged to the oEGL and 35 (58%) to the iEGL. On the other hand, 8 (73%) of the prometaphase cells belonged to the oEGL and 3 (37%) to the iEGL (Fig. 2C). Telophase cells were located at the boundary between the oEGL and iEGL (Fig. 2C). These latter observations indicate that GCs divide in not only the oEGL as previously thought (*26*) but also the iEGL (*27*). Furthermore, IC-connected cells substantially outnumbered GCs in prometaphase and telophase, challenging the possibility that all ICs are mitosis-derived CBs. We therefore decided to study the morphological features of IC-connected cells and explore whether these characteristics could shed light on their origin and function.

### Morphology of connected GCs differentiate between ICs and CBs

**Fig. 3.**
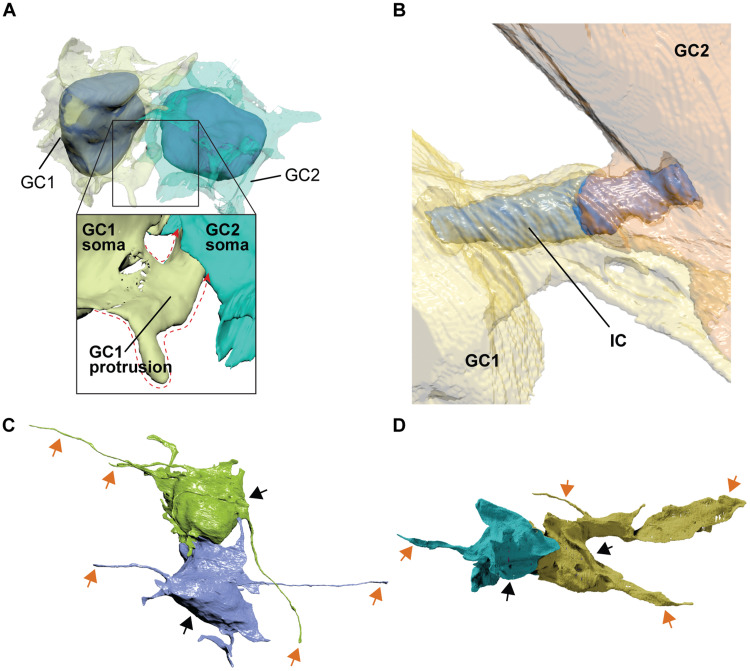
Morphological assessment of IC-connected GCs reveals distinct cellular features. (**A**) 3D reconstruction showing an example of IC-connected GCs via a cell protrusion (protrusion contour depicted by red dotted line): GC1 and GC2 connected via IC originating from protrusion of GC1. (**B**) 3D reconstruction showing sheets wrapped around IC (blue) extending from both connected cells (GC1, yellow; GC2, orange). (**C** and **D**) 3D reconstruction of IC-connected GCs (cell somas, black arrows) extending filopodia and lamellipodia (orange arrows).

By using 3D reconstructions of fully segmented IC-connected cells, we revealed three intriguing morphological features that may differentiate ICs from classical CBs. First, in contrast to the stereotypical CB that connects the soma of two cells and aligns predominantly with the nuclei of daughter cells (*18*), some of our ICs connected to other parts of the cell, at times even connecting a process of one cell to the soma of the other, rather than simply connecting soma to soma in an aligned fashion (Fig. 3A). Second, ICs were not always directly exposed to the extracellular matrix, as is the case for CBs in vitro (*20*) and in vivo (*28*). Instead, they were wrapped by membranous lamellipodia-like sheets that stemmed from both connected cells (Fig. 3B). Third, in several cases, IC-connected GCs extended protrusions of up to 31.15 μm in length (Fig. 3, C and D), which is an unexpected behavior of dividing GCs as membranous processes are thought to develop after cells disconnect from each other after cytokinesis (*27*). Although GCs are expected to have extended parallel fibers by the end of their tangential migration, GCs with protrusions being connected makes this observation unique.

### IC-connected GCs are mitotic, migrating, or intermediate

**Fig. 4.**
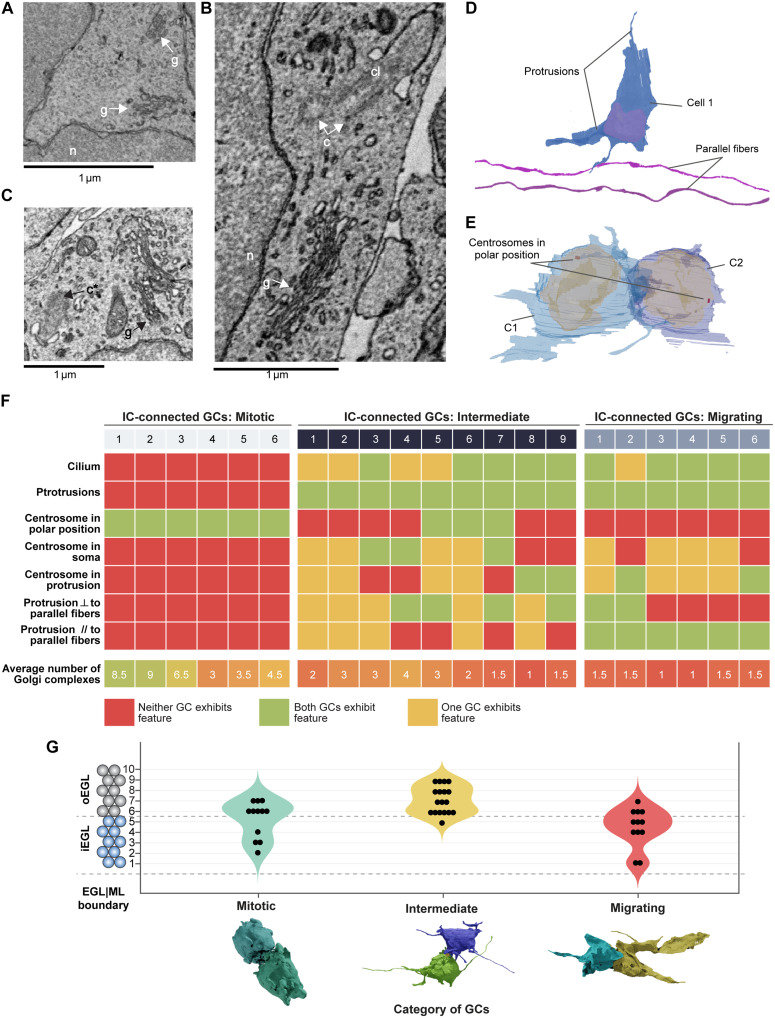
IC-connected cells are mitotic, migrating, or transitioning between the two states (intermediate). (**A** to **C**) Identification of cellular organelles within GCs in electron micrographs: n, nucleus; c, centrosome; g, Golgi complex; c*, centriole forming a cilium; cl, cilium. (**D**) GC showing protrusions and two parallel fibers (purple) as a reference for orientation; the protrusion orientation was described with respect to the parallel fibers. (**E**) IC-connected GCs showing centrosomes at polar positions indicating a mitotic stage of GCs. (**F**) Summary of features based on cellular organelles and protrusions for three categories of IC-connected GCs: mitotic, intermediate, and migrating. Total 42 GCs (21 ICs) considered for this analysis. ⟂ stands for perpendicular, // stands for parallel. (**G**) The distribution of IC-connected GCs (*n* = 42) in three categories: mitotic (*n* = 12), intermediate (*n* = 18), and migrating (*n* = 12).

To determine whether ICs could have resulted from cell division, we investigated the cell-cycle stages of IC-connected GCs. This was inferred morphologically by 3D reconstruction of cell shape and characterization of subcellular features that change during different stages of the cell cycle. These included the number and distribution of Golgi complexes, the position of centrosomes, and the presence of a primary cilium (Fig. 4, A to C, and fig. S3). We selected 21 IC-connected GC pairs for this analysis (i.e., 42 GCs), specifically because all morphological features in consideration were visible in the volume.

Our analysis revealed three major categories of IC-connected GC-pairs: mitotic, migrating, and intermediate (fig. S3). Of these, 6 GC pairs (12 GCs) were mitotic. These cells had more than two Golgi complexes dispersed in the cell (Fig. 4, D and F, and fig. S3, A and B) and were characterized by the absence of a cilium, both features of dividing cells (*29*–*31*). Accordingly, these cells were spheroid in shape and had centrosomes positioned at opposite cell poles, probably corresponding to spindle poles (Fig. 4, E and F, and fig. S3) (*32*). Together, these observations suggest that these cells may be at the end of mitosis, presumably in late telophase/early cytokinesis (*33*).

Another 6 pairs (12 GCs) were migrating. These cells harbored fewer than two Golgi complexes and bore a cilium, indicating a G_0_/G_1_ stage (Fig. 4F, and fig. S4, A and B). They also showed well-developed lamellipodia oriented parallel to the EGL/ML boundary and parallel fibers, a characteristic of tangential migration (Fig. 1A and fig. S4) (*34*). Most of these GC pairs were found in the iEGL, consistent with their tangentially migrating stage.

On the basis of the morphological criteria analyzed above, we could not categorize the remaining nine pairs as undergoing migration or cell division and were therefore defined as “intermediate” (Fig. 4F). These cells were not “truly” migrating tangentially as their protrusions did not show any specific orientation (Fig. 4D) and they were mostly located in the oEGL (Fig. 4G). These GCs exhibited diverse shapes; for instance, one pair showed cells with a round soma and long filopodia, while two other pairs had elongated somas and small lamellipodia. Intermediate cells also shared morphological features with mitotic and migrating cells. For example, some had more than two Golgi complexes and a cilium. In these cells, the centrosome was mainly located in the soma (not close to a protrusion), supporting the fact that they were not undergoing migration. Thus, this analysis suggests an overlap of events at the end of mitosis and extension of protrusions (Fig. 4F).

Two mitotic GC pairs were located deep in the iEGL (Fig. 4G), reinforcing the observation that cell division occurs in not only the oEGL as previously proposed (*26*) but also the iEGL. Similarly, 4 of 12 migrating GCs were observed in the oEGL, implying that migration is not restricted to the iEGL (Fig. 4G).

In 8 of 12 migrating GCs, the centrosome was located close to one of the protrusions, as expected for migrating neurons, where centrosomes have been shown to be in the vicinity of the leading process or a lamellipodium (*35*, *36*). For the remaining four migrating GCs, the centrosome was in the soma, and for two of these GCs, also close to the IC. These observations could imply that the centrosome was no longer in charge of spindle poles, and GCs in the migrating category have exited mitosis.

### ICs are anatomically diverse and complex

**Fig. 5.**
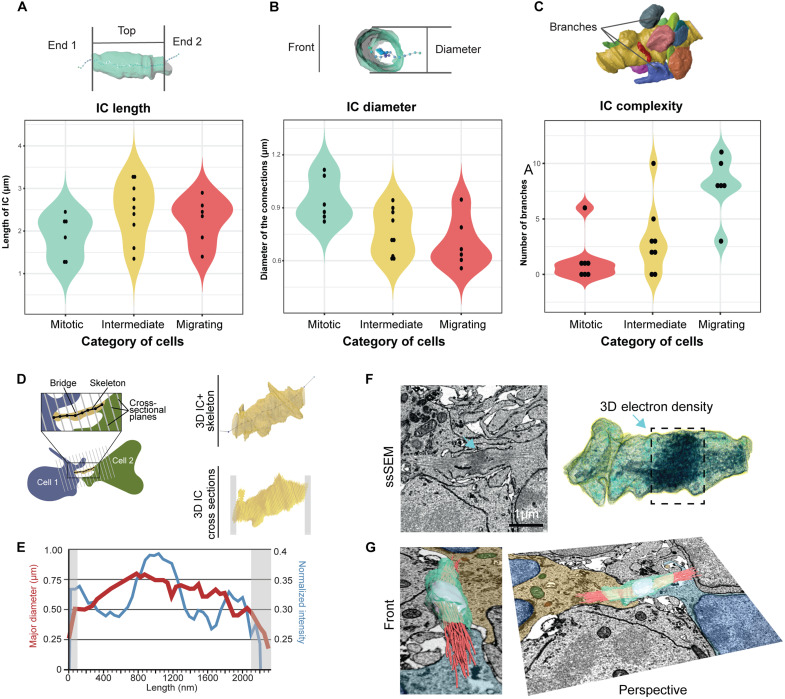
Morphologic characterization of the connection in interconnected GCs. (**A**) 3D reconstruction of an IC with a skeleton. End 1 and end 2 mark sites of contact with GCs, depicting the boundaries of the length calculation of ICs. (**B**) IC with a skeleton from front view showing diameter. (**C**) Complexity of IC (yellow) determined by the number of branches. (**D**) Schematic showing method used to slice IC into cross sections along its length, assisted by a Kimimaro skeleton, with a 3D reconstruction of IC (right, top) and resulting 50-nm cross sections (right, bottom); gray boxes indicates unusable sections. (**E**) Line plot depicting the major diameter (red) and normalized pixel intensity (blue) distributions along IC length. Gray zones indicate unusable sections shown in (D) that were not considered for analysis. (**F**) Section from ssSEM volume (left) and 3D electron density reconstructions (right) showing the electron densities along an IC graphed in (E). (**G**) 3D reconstruction of (F) shown from two angles, with reconstructions of internal densities (MB in cyan and microtubules in red), overlayed on a ssSEM slice containing the two GCs connected by IC.

To characterize the diversity of ICs, we developed a program for morphometric analysis of segmented cells called CellWalker that allows for calculation of morphological features of segmented cellular structures, including length, diameter, volume, surface area, and curvature (see Supplementary Methods). Using CellWalker, we saw that IC lengths ranged from 1.25 to 3.3 μm with an average of 2.25 μm (Fig. 5A), comparable to in vitro and in vivo CBs, which exhibit lengths between 3 and 5 μm (*37*).

To assess trends in the thickness (or diameter) of ICs, we sliced each IC at 50-nm intervals along their lengths to obtain a series of cross sections (Fig. 5, B and D). To assist the determination of the axis for cross sectioning, we skeletonized ICs using the Kimimaro algorithm (*38*) (see Supplementary Methods). We observed that cross sections were largely circular, as evidenced by their elongation values (fig. S5), ranging between 0.14 and 0.3 (average value 0.22). This circularity allowed us to compare cross-sectional diameter profiles of ICs, revealing a range of average diameters between 0.56 and 1.12 μm, in agreement with in vitro CB measurements (Fig. 5B) (*37*).

We then compared lengths and diameters of mitotic, migrating, and intermediate cell categories. IC lengths of mitotic cells ranged between 1.3 and 2.25 μm, compared to 1.85 and 2.6 μm in migrating cells (Fig. 5A), indicating that connections can stretch longer in migrating cells than in mitotic ones. The length of connections between intermediate cells ranged from 1.6 and 3.25 μm.

Conversely, the connection diameter appeared larger in mitotic cells (from 0.85 to 1.082 μm) than in migrating cells (from 0.604 to 0.789 μm), with intermediate cells distributed mostly between mitotic and migrating cells (Fig. 5B).

The morphological complexity of ICs was also analyzed. Unlike previously described membranous blebs adjacent to CBs (*20*), several ICs had membranous protrusions (branches) (fig. S4, B′ and B″) that shared their lumen with the shaft of the connection. Most ICs in mitotic cells had 8 to 10 branches, compared with 1 or 0 branches on the ICs of migrating GCs. The complexity of ICs on intermediate cells seems to be in between mitotic and migrating cells, with most having two to five branches (Fig. 5B).

One of the most peculiar features observed within ICs were electron-dense regions, which we presumed to be microtubule bundles (*18*, *39*). We quantified the overall voxel intensity in each cross section along ICs as a proxy for cytoskeletal filament density (Fig. 5, D to F, and fig. S6). Profiles obtained from intensity-scaled electron micrographs showed that ICs contained dense regions that could correspond to MBs (Fig. 5F). Segmentation of darker voxels and 3D navigation using the virtual reality software, DIVA (*40*), illustrate these quantitative findings and provide a realistic representation of the cytoskeletal architecture within connections (Fig. 5G).

We also identified several vesicular and membranous compartments within ICs. Segmentation of these compartments revealed volume and morphology heterogeneity, ranging from small spherical vesicles to large extended compartments (fig. S7). The latter have not been found inside typical CBs. Overall, the cargos occupied a small fraction of IC volumes and were distributed throughout the bridge; however, the central dark intensities of ICs could also have obscured cargos in this area, meaning that the true cargo presence is greater than we could detect.

## DISCUSSION

### Overview of the EM volume and segmentation

The complex properties of the adult mammalian brain arise from a finely orchestrated series of neurodevelopmental processes (*8*). Abnormal laminar cytoarchitecture and cortical disorganization of neurons can lead to severe developmental disorders (*41*).

To better understand the maturation process of dividing and migrating progenitor cells in the developing brain, we focused on cerebellar GCs located in the transient EGL for their ability to proliferate and migrate in a continuous, rolling fashion, and whose multiple stages of maturation into neurons can be studied using a 3D ssSEM snapshot all at once (*14*, *42*). Intriguingly, we found that both oEGL and iEGL contained GC pairs connected through ICs in the oEGL and iEGL.

Given that the oEGL at of the cerebellum of mice in postnatal stages harbors proliferating GCs, it is conceivable that some ICs correspond to CBs. The observation of GCs in prometaphase and telophase located in the iEGL suggests that GCs do not follow a deterministic maturation between EGL sublayers but rather, that they may be able to enter a mitotic state while in the iEGL. This discovery offers a perspective to the study of the developing EGL and raises the question of whether dividing cells in different laminar layers have different precursors and/or whether they follow the same cell fate.

### IC-connected GCs exceed the expected number of GCs from prometaphase cells and show diverse morphology

Intriguingly, the total number of connected cells that the 11 cells in prometaphase could have generated (i.e., 22) did not match the number of IC-connected GCs (i.e., 60). If the 30 ICs were in fact CBs, an explanation could be that cytokinesis in GCs takes longer than mitosis. Another is that may be that at least some GCs deliberately retain their CBs to keep cells attached in a manner similar to IBs, stable CBs that form as a result of incomplete cytokinesis in spermatogonial cells (*43*, *44*). IBs could serve as a scaffold to transport key proteins to control cell polarization (*39*) or synchronize future divisions (*23*). The observation of different cargoes, including membranous compartments and mitochondria, within ICs might support this hypothesis, as persistent IBs represent a mechanism of intercellular transport distinct from conventional vesicle trafficking in MBs of CBs.

Given the fixed time point constraint of our dataset, confirming the source of the unequal ratio of prometaphase and IC-connected cells is challenging. However, the fact that we observed neither cells interconnected as syncytia (i.e., more than two connected cells in a row) nor microtubule-bare ICs, both of which are features observed in IBs (*6*, *44*), suggests that many of the ICs we observed differ from IBs previously described in gonad maturation (*6*).

Our 3D reconstructions revealed three specific features that differentiate ICs from classical CBs, each of which could give clues about the nature of ICs. First, IC-connected GCs that are connected at locations other than their somas may indicate a time lag that gave CBs the opportunity to shift in position (Fig. 3A). Second, ICs were wrapped by membranous, lamellipodia-like sheets that stemmed from both connected GCs (Fig. 3B). Enveloping sheets could potentially play a role in protecting membrane remodeling and thereby in stabilizing the bridge. Alternatively, extended sheets could be in the process of engulfing the MB, which was proposed to be an intracellular signaling organelle that regulates cell proliferation (*45*). Third, some IC-connected GCs in the iEGL have long (up to 30 μm) protrusions, suggesting that ICs (if they originate during cell division) may stay connected for a considerably long time after mitosis ends (Fig. 3, C and D). GC protrusions have been considered to be precursors of parallel fibers, which immature GCs extend into the ML as they migrate down to the inner granule layer (*46*). This interpretation would suggest that our connected cells with protrusions are in the process of migrating out of the EGL.

### IC-connected GCs: Mitotic, intermediate, and migrating

The above observations indicate that at least some of these connected GCs exhibit characteristics unexpected for conventional mitotic cells. We grouped connected GCs into three categories, mitotic, intermediate, and migrating cells, using several cytoarchitectural features. On the basis of this categorization, several inferences can be drawn about the behavior of each type of IC-connected GC.

All migrating IC-connected GCs have processes whose orientation follows that of parallel fibers', which indicate tangential migration, but some of them also show protrusions perpendicular to parallel fibers and centrosomes located close to these processes. Centrosomes in migrating cells are observed at different locations: in the soma or near the protrusion, which may suggest different stages of migration.

Connections between migrating cells could be remnants of mitosis or formed de novo. Dubois *et al.* (*47*) described that the centrosome was implicated in tunneling nanotube (TNT) formation and that the Golgi complex was positioned in front of the connection, with the centrosome behind it. In addition, the centrosome has also been reported to be oriented toward TNTs. In migrating, IC-connected GCs, we observed this specific arrangement of the centrosome and the Golgi complex with respect to the connection for 7 of 12 GCs. In four of these, both the connected cells presented one centriole pointing towards the connection.

Intermediate GCs could then be considered as being chronologically located between mitosis and migration, following the hypothesis of a continuum of events represented by the presence of mitotic, intermediate, and migrating cells. Such intermediate cells have been described as arising from asymmetric division and expressing markers of GC progenitors (GCPs; MATH1), and early postmitotic and tangentially migrating cells (Doublecortin,) (*48*). Furthermore, GCs in the developing chick cerebellum have been shown to grow and retract protrusions in between proliferative events, which could indicate that protrusion extension in GCs is not directly correlated with cell fate (*27*).

### Morphology of connections correlates with the category of GCs

The profiles of pixel intensity in the electron micrographs along connections provided an arbitrary proxy of the microtubule-like filaments running within ICs. Such bundles of microtubules are well known in CBs, and they form the MB in the CB, along with several other proteins and vesicular cargoes. We also observed various vesicular bodies forming cargo inside ICs (fig. S7), which corresponds with the vesicles shuttled by the microtubules within CBs for the completion of abscission in dividing cells (*49*). Our observation of a mitochondrion inside one of the ICs is perhaps indicative of their dissimilarity from conventional CBs.

Furthermore, trends in length, diameter, and complexity may indicate the changes in the morphology of these supposedly persistent CBs as the dividing cells progress toward migration (Fig. 5). We can imagine that it would be easier for two cells to migrate together, being connected by longer, thinner, and less complex structures, notably in a compact tissue environment.

### Protrusion growth takes longer than mitosis

Last, our IC connected GCs have long lamellipodial protrusions ranging up to 31.15 μm with an average length of 9.5 μm. The time-lapse imaging performed by Hanzel *et al.* (*27*) in the developing chick cerebellum suggests that a protrusion of 29.14 μm would take ~220 min to grow. At this speed, the longest protrusion of 31.15 μm in our connected GCs would require ~235 min, while an average 9.5-μm-long protrusion would take ~72 min to grow. These durations are nine and three times longer than the average time of mitosis, ~25 min (*50*, *51*), respectively. These results make us rethink our vision of individual cells undergoing well-separated metabolic events.

### Limitations and speculations

The trade-off from investigating the cerebellar cortex using ssSEM, a technical-, time-, and financially demanding methodology, resulted in our full characterization being limited to one animal and one time point. Hence, our results may vary animal to animal (including male versus female), vermis-to-hemispheres regions, and lobule to lobule. Given that the hierarchical architecture of cellular types and layers in the cerebellar cortex is the same for all lobules and both vermis and hemispheres (*52*), we expect that our major observation, the existence of ICs, also applies to them. However, it is likely that the number of ICs per lobule may not be consistent across all lobules, as the thickness of the EGL (and therefore, the number of GCs per lobule) was shown to vary slightly between lobules at comparable developmental stages (*46*). Although the assessment of our other two volumes (P0 and P3) is still preliminary, it supports our hypothesis that ICs are not subject to one animal or one developmental stage. Deciphering the molecular makeup of ICs will enable future studies to address the limitations of our study by using alternative methods such as immunohistochemistry and fluorescence-based microscopy.

While the limitations of our ssSEM-based approach do not allow us to explore the dynamics and biogenesis of cytoplasmic ICs between GCs, this technique provides an ultrahigh-resolution view of the EGL of a developing mouse cerebellum. We believe that ICs were never observed in the past since this is the first time a high-resolution 3D reconstruction approach was used to study GCs in the EGL. Detailed morphological characterization of these GCs reveals that their development in the EGL is more diverse and complex than conventional descriptions indicate. The wide range of features exhibited by ICs highlights the morphological diversity across connections and connected GCs. Our analysis indicates convincing signs of mitotic, migrating, and intermediate stages of connected cells, as evidenced by the presence and arrangement of organelles such as the Golgi complex, centrosomes, cilia, and cellular protrusions. ICs may be mitotic in origin, owing to their characteristics, such as microtubule bundles, presence of MB-like densities, and dimensions similar to CBs. The hypothesis that ICs originate from mitosis suggests their persistence until the maturation of protrusions. Our rate estimations of protrusion growth suggest that GCs remain connected for several hours after mitosis. Hence, irrespective of their origin, the observations of cells with long protrusions being connected may indicate that they are connected for purposes such as transfer of vesicles, mitochondria, and molecules between cells, as has been demonstrated in case of tunneling nanotubes (*53*–*55*). Recent results from Dubois *et al.* (*48*) might also suggest that in some connected GCs, the centrosome close to the opening of the IC may be involved in their translocation through these connections or help in the formation of the de novo ICs. Overall, our results open several avenues of research to investigate the origin and function of ICs between developing GCs. Future research in living tissue may be required to diagnose the mechanisms underlying their formation and physiological function during cerebellar development, as well as assessing whether this phenomenon is widely present and relevant for CNS development.

## MATERIALS AND METHODS

### Datasets

All animals were handled according to protocols approved by the Institutional Animal Care and Use Committee at the Harvard University.

### P0

An unsexed, P0 CD1 wild-type mouse was anesthetized by hypothermia. The brain was harvested and postfixed in paraformaldehyde/glutaraldehyde solution overnight at 4°C O/2 N. Then, the cerebellum was cut into 300-μm-thick parasagittal sections using a Leica vibrating microtome. The cerebellum was vibratomed in ice-chilled cacodylate buffer solution. Sections from the medial part of the vermis were stained using reduced osmium-thiocarbohydrazide (TCH)–osmium (ROTO). The ROTO protocol was used to improve membrane contrast further by double labeling with osmium, using TCH as a link molecule. First, sections were incubated in 2% osmium tetroxide in potassium ferrocyanide (0.015 g/ml) in 0.15 M NaCaCo, 2 mM CaCl_2_ buffer at 4°C overnight. Then, the sample was transferred to the buffer solution for five 2-hour changes and incubated in 1% TCH in buffer that had been heated to 60°C for 20 min. After washing the tissue again with buffer as described above, the tissue was incubated in 2% osmium tetroxide in buffer at 4°C overnight. After staining and postfixing with ROTO, we dehydrated the tissue by washing it in solutions of increasing ethanol content (5 min each in ice-cold solutions of 20, 50, 70, 90, 100, and 100% ethanol in ddH_2_O) and last, by placing the tissue in anhydrous acetone left at room temperature for 10 min. We then embedded the tissue in Epon 812 resin mixed according to formula that would result in a medium-to-hard cured product (Electron Microscopy Sciences). We embedded each section in a block of resin by introducing solutions of propylene oxide (PO). Using mixtures of PO and resin thus allows the resin to better penetrate spaces in the tissue. We washed each section two times for 5 min each in pure PO, then left it in 3:1 PO/resin solution under constant agitation for 1 hour, followed by 1:1 PO/resin under agitation overnight, then 1:3 PO/resin under agitation for 1 hour, and then in 100% resin overnight. Last, we embedded each section in a tube of fresh resin and cured the block at 60°C for 48 hours.

Next, from each block of embedded tissue, we cut a series of 30-nm-thick serial sections for imaging using an automatic tape-collecting ultramicrotome (ATUM). The ATUM combines an ultramicrotome with a reel-to-reel tape-collecting system so that sections can be continuously cut and collected on the tape with minimal user intervention. The ATUM allows many consecutive sections to be cut, collected onto a reel of tape, and then imaged at 4 nm/px (or 4 × 4 × 30 nm^3^ per voxel, where 30 nm is the distance between two consecutive sections). Image acquisition, stitching, and alignment of the volume were carried out as previously described (*14*). The aligned and stitched image volume comprised 1450 sections imaged.

### P3 and P7

The P3 and P7 datasets used in this study were obtained from the medial part of the cerebellar vermis in lobule VIII of unsexed, CD1 wild-type P3 and P7 mice pups, previously published (and available online at https://bossdb.org/project/wilson2019). The aligned and stitched image volumes contained sections cut in the sagittal plane and imaged at 4 nm^2^/px resolution (or a anisotropic voxel size of 4 × 4 × 30 nm^3^). The preparation of this volume was carried out as described above, and as previously published (*14*). Note that for all calculations of morphological features, the downsampled data at lower resolution (i.e., 32 × 32 × 30 nm^3^ per voxel) was obtained using the VAST software package to reduce computational cost.

### Identification of cellular features in the ssSEM volume

#### Cerebellar GCs

Identification of GCPs in the EGL was performed manually based on GCP location, size, morphology, and nuclei features: The location of GCPs was determined by defining the boundaries of the EGL (see the “Reconstruction of the EGL|ML boundary” section below). At their widest point, the somas of GCPs had diameters of ~7 μm and exhibited a round-shaped body. GCPs contained nuclei that occupied most of the cytoplasm. Mitotic cells: GCPs in prophase were primarily distinguished by their disassembled nuclear envelope. The disassembled nuclear envelope was observed in the ssSEM volume as a fragmented membrane in the cell’s cytoplasm. Cells in prophase also exhibited a spherical shape and lacked protrusions. GCPs in telophase were identified on the basis of a wide cleavage furrow that bridged the cytoplasm of two spherical-shaped cells; the nuclear envelope of both cells was already assembled. ICs were identified on the basis of a cylindrical tube shape connecting the cytoplasm of two cells; the presence of an electron-dense region resembling a MB and microtubules emanating from the potential MB-ring into both cells. GCPs connected by ICs had fully formed nuclei. Tentative MBs were identified by a cluster of electron dense voxels inside the IC and predominantly between microtubule filaments on each side. Microtubule filaments were identified on the basis of their electron density, straightness and fineness, and thickness (~25 nm). Cargoes within ICs were identified by membrane enclosure. Spherical cargoes included vesicles; nonspherical cargoes were labeled as membranous compartments. Mitochondria were distinguished by their shape and cristae. Protrusions extending out from the IC were classified as IC branches. Classification of branches was performed manually based on their 3D shape.

### Checks on dataset usability

Initial identification of cells in the EGL was performed manually via VAST (https://lichtman.rc.fas.harvard.edu/vast/) by annotating the nucleus of every cell (or cell soma for cells with disassembled nuclei). Slight shifts in *x* and *y* during image acquisition of the dataset prevented exhaustive use of cells located at the top of the EGL, including all cells bordering the pia layer and cells on both side edges. To accurately estimate the number of cells in the volume, we employed the following rule: (i) classify cells as either (a) containing a fully visible nucleus ITV and (b) partially visible OOV; (ii) all cells ITV within a distance range of 7.5 μm from the center of the cell (the average thickness of a GCP) were flagged as likely OOV. Of 2042, this method yielded an estimate of 932 GCPs.

### Tracing and rendering of ssSEM data

Regions of interest were manually traced with VAST. For rendering of all the traced images, 3D surface meshes of labeled objects were generated from VAST using VastTools (written in MATLAB) and imported into 3D Studio Max (Autodesk Inc.) to generate 3D renderings of all the traced objects.

### Cell-level distribution analysis

The positions of mitotic and IC-connected GCPs along the radial axis in the EGL were assigned manually by counting GCPs, starting from GCPs bordering the ML and ending at GCPs nearing the pia. GCPs in levels 1 to 5 (1 being the layer closest to the ML) were classified as iEGL cells and GCPs in levels 6 to 10 (10 being the layer closest to pia) were classified as oEGL cells.

### CellWalker

The software CellWalker is scripted in Python (tested on version 3.x) and is available freely under the GNU General Public License (version 3.0) (https://github.com/utraf-pasteur-institute/CellWalker). Dependencies are described in the user manual (https://github.com/utraf-pasteur-institute/CellWalker). CellWalker supports image sequences of segmented microscopy images in PNG format. CellWalker exports various types of outputs depending on the analysis performed, which include skeleton .swc files, .csv files for results of characterization, and Wavefront OBJ files for 3D visualization. These OBJ files can be directly imported in 3D rendering software Blender.

### Blender-Python script for cross sectioning the objects

This script is provided along with the CellWalker software. The usage is described in the manual (https://github.com/utraf-pasteur-institute/CellWalker/tree/main/src/blender_python_scripts).

### Calculation of elongation of cross sections

Elongation value was calculated for each cross section along the connection to confirm the circularity of the cross section. Elongation of a 2D shape can be calculated as follows

Elongation=1−(minor diameter/major diameter)

The elongation ranges between 0 and 1, with values close to zero indicating circular shapes (see fig. S5). The ICs in our data showed elongation values ranging between 0.14 and 0.3 (average value, 0.22), indicating that the ICs were fairly circular in cross section. Therefore, we chose major diameter as the diameter of the ICs.

### Cross sectioning of IC along length

The ICs were cross sectioned along their lengths using in the Blender software, which works on the OBJ meshes exported from VAST or CellWalker. The cross sectioning was assisted by a skeleton built using Kimimaro algorithm. The Kimimaro skeleton was built for an IC by loading its segmentation (exported from VAST) into CellWalker. CellWalker’s skeletonization module helps applying the Kimimaro algorithm on selected segments in the loaded segmented image stack. The skeleton was visualized inside the CellWalker to manually identify the nodes on the skeleton that would form an axis approximately along the length of the connection. CellWalker’s ability to export skeleton as OBJ file allowed us to load it into Blender and confirm that the selected nodes indeed formed an axis along the length of the IC. This axis (also called “centerline”) was also exported by CellWalker as an OBJ file. Next, the OBJ file of the IC (from VAST) and OBJ file for the centerline (from CellWalker) were loaded in Blender. A Blender-Python script (provided in the GitHub repository above) was written to slice the mesh of the IC along the centerline at each point on the centerline (50-nm spacing decided by CellWalker). The Blender-Python script also calculates the properties of the slices (or cross sections), including major axis, minor axis, and cross-section area (more features can be found in the script).

### Scaling and normalization of voxel intensities

All intensity values of the voxels were inverted, so that higher values correspond to darker regions in the ssSEM images. This design choice was made to facilitate the interpretation of our measurements of cytoskeletal density, since in this case, the elements of interest are electron dense.

To allow for comparison of cytoskeletal element density at bridges located in different regions of the ssSEM image volume, where pixel intensities may vary, we then min-max scaled intensities as follows

$$I_{\left(i,\mathrm{scaled}\right)}=\frac{I_{i}-I_{\min}}{I_{\max}-I_{\min}}$$

where “*I*” stands for intensity.

The sum of voxel intensities in a given slice was then obtained as a weighted average of the whole slice (see fig. S6). A voxel intensity profile along an IC was created by generating a centerline (described in previous section) along the connection and making cross sections at 50-nm interval along the center line. The inverse voxel intensities are used to calculate a weighted average for each cross section to generate the intensity profile along the connection. The profiles of this electron density along all our ICs showed a peak within the IC corresponding to the region where darker microtubule-like bundles overlap (example shown in Fig. 5, C and D). This peak also corresponds with a slightly increased diameter along the IC length (see Fig. 5C).

### Reconstruction of the EGL|ML boundary

The EGL|ML boundary is necessary as a reference for calculation of orientation of the connected cells. Conventionally and intuitively, the pia membrane is used for this purpose. However, in our ssSEM data, the pia is incomplete. The segmentation of the available traces of the pia indicated that it would be insufficient for calculating orientations of connected cells in the full volume. Therefore, we made use of the EGL|ML boundary as a proxy for the pia.

Manual identification of the ML through the entire data volume is an extremely time-consuming and intensive process. To make the process faster and reproducible, we designed a computational pipeline using deep learning classification followed by 3D point-cloud processing.

In short, the full pipeline can be described as follows. A CNN classifier was trained to detect cytological features to differentiate cells in the EGL from parallel fibers in the ML and was then applied to uniformly sampled image tiles (300 × 300 pixel^2^) from the entire ssSEM volume (fig. S1). The training dataset consisted of randomly chosen and manually labeled EGL and ML tiles. In the inference phase, the CNN was applied to uniformly sampled tiles from the full ssSEM volume. We used the coordinates of image tiles that were identified by the CNN as ML to create a point cloud and applied a ball-pivoting algorithm to construct a mesh demarcating the EGL|ML boundary. We then applied a cubic spline interpolation to remesh this coarse-grained surface and obtained a dense 3D surface (shown in light yellow in Fig. 2A). The detailed procedure is as follows.

### Training data

The resolution (mip) level 3 data were used for training the CNN. At mip level 3, the pixel resolution is 32 × 32 nm^2^ per pixel. Four-hundred tiles of size 300 × 300 pixel^2^ were randomly chosen from the ssSEM volume representing both EGL (200 tiles) and ML (200 tiles) classes. These tiles were subjected to augmentation to generate a larger training dataset as follows.

### Train/test split

Each selected region was used to generate several augmented tiles. We split-training images into train and test datasets before augmentation to ensure that augmented images from the same tile do not end up in both the train and test datasets. We split the 200 tiles of each EGL and ML class as 160 tiles in the train dataset and 40 tiles in the test dataset, i.e., 80:20 proportion.

### Data augmentation at source

For each class (EGL and ML),

{

 For each tile

 {

  Apply shift in X and Y (Shift: 300 px at mip0 = 300/8 pixels at mip3)

  Original tile + 0 px shift

  Original tile + 300 px shift in X

  Original tile - 300 px shift in X

  Original tile + 300 px shift in Y

  Original tile - 300 px shift in Y

  Resultant number of shifted tiles = 5

  For each shifted tile

  {

   Apply rotations: 0, 30, 45, 60, 90 degrees

   Resultant number of tiles = 5

  }

 }

}

Total numbers of augmented tiles are as follows: For EGL class, 200 × 5 × 5 = 5000 (train: 4000 and test: 1000); and for ML class: 200 × 5 × 5 = 5000 (train: 4000 and test: 1000)

### Manual filtering

These 5000 tiles belonging to each class are manually filtered to remove blank and partially out-of-volume tiles as well as ML tiles that contain portions of GCs.

For EGL class after filtering: 4875 tiles (train: 3900 and test: 975)

For ML class after filtering: 3400 tiles (train: 2800 and test: 600)

The total number of manually filtered tiles is thus 8275 with 6700 (81%) tiles in the train dataset and 1575 (19%) tiles in the test dataset.

### Training/validation split

Train dataset was split with a 70:30 proportion to obtain training and validation images before feeding to the CNN classifier.

### Stochastic augmentation (applied during the training process)

Left-right flipping (mirror operation) with 0.5 probability

Random brightness (max_delta = 0.4)

Random contrast (lower limit 0.2, upper limit 0.6)

### CNN architecture

The CNN architecture was designed as illustrated in fig. S1. Our CNN has three convolutional layers with ReLU activation (kernel size 3 × 3) of sizes 32, 32, and 64, respectively, with a 2 × 2 max-pooling layer followed by each. The “same” padding was used in all convolutional layers. The output of the convolutional block is flattened and a 50% dropout is applied as a measure against overfitting. After the 50% dropout layer, two dense layers with ReLU activation are inserted with 512 and 128 nodes, respectively. Each dense layer is also followed by a batch normalization layer. In the end, a dense layer with a single node with a sigmoid activation is inserted to obtain the predicted value (0, EGL; 1, ML). We used an ADAM optimizer with a learning rate of 0.0001. The loss metric was binary cross-entropy.

The model with the smallest loss was chosen. Because of the clearly differentiable images of EGL and ML, the resulting model was extremely accurate, achieving F1 scores of 0.9989 and 0.9983 for EGL and ML, respectively, on the test data. The confusion matrix showed only two mis-classifications (of 1575 total) where an ML image was classified as an EGL image (fig. S1).

### Inference (applying the classifier on the ssSEM volume to identify EGL and ML)

The tiles of size 300 × 300 pixel^2^ were sampled uniformly from the ssSEM volume with spacing of 100 px, 100 px in *x* and *y* directions, and 100 slices in the *z* direction (at mip level 3), which correspond to approximately 3-μm spacing. A total of 15,636 tiles were sampled such that they covered both EGL and ML. The trained CNN classifier was applied to these tiles and a cutoff of 0.9 was used to threshold the prediction score. This threshold ensured the correct identification of the ML tiles.

### Implementation of the CNN pipeline

The CNN pipeline was designed using TensorFlow-keras (version 2.4). Training was performed on a Kaggle (https://kaggle.com/) kernel with Tesla P100 GPU. The training process was fast (9 s per epoch). Training batch size was set to 64 for optimal use of the GPU.

Inference was performed on a local computer (Dell Precision 3000 workstation with Intel Core i9-9900 processor and equipped with Nvidia Quadro RTX4000 GPU). Prediction on 15,636 tiles sampled from the ssSEM took 6.8 s. The batch size was set to 64.

Our code is available as IPython notebooks on GitHub (https://github.com/utraf-pasteur-institute/ssSEM-EGL-ML-analysis). Datasets are available as indicated in the Data and materials availability section.

### Reconstruction of EGL|ML boundary surface using CNN predictions

The center coordinates of the tiles identified as ML tiles by the CNN classifier were used to create a point cloud using a Python Open3D library. The normals were assigned to each point in the point cloud such that they oriented toward the EGL. The ball-pivoting algorithm (as implemented in the Open3D library) was then applied to generate a mesh from the point cloud. The assignment of normals facing in the direction of the EGL was necessary to ensure that the ball-pivoting algorithm identifies the required orientation in the point-cloud. The assignment of normals took care of the correct facing of the surface mesh such that it was created at the EGL|ML boundary.

This mesh was then exported as a .ply file (Stanford format) and loaded in the 3D rendering software Blender for visualization and manual fine-tuning. The fine-tuning was necessary because of the anomalies in the ssSEM data such as badly imaged regions (scratches, dark/light patches, etc.), GCs passing through the ML, and also the nature of the EGL|ML boundary, which is extremely uneven in certain regions. These anomalies led to the generation of holes in the EGL|ML boundary surface that needed manual attention. The fine-tuning step also involved deleting all other points in the point cloud that did not contribute to the EGL|ML boundary.

After closing the holes in the reconstructed EGL|ML boundary and removing unwanted points, we performed a remeshing operation using cubic-spline interpolation implemented in 3DSmax software. The resulting dense mesh (that contained approximately 54,000 points) representing the EGL|ML boundary was used for all further analysis. The code is available as an IPython notebook on GitHub (https://github.com/utraf-pasteur-institute/ssSEM-EGL-ML-analysis).

## Notes

View/request a protocol for this paper from *Bio-protocol*.
