# Supplementary material for: 3D reconstruction of the cerebellar germinal layer reveals tunneling connections between developing granule cells
Source: Sci Adv. 2023 Apr 5;9(14):eadf3471. doi: 10.1126/sciadv.adf3471 (PMC10075961; doi:10.1126/sciadv.adf3471)
Supplement: Supplementary file 1 — Figs. S1 to S7 Legends for movies S1 and S2 [file sciadv.adf3471_sm.pdf]

Science Advances
AAAS

Supplementary Materials for

3D reconstruction of the cerebellar germinal layer reveals tunneling connections between developing granule cells

Diégo Cordero Cervantes *et al.*

Corresponding author: Chiara Zurzolo, chiara.zurzolo@pasteur.fr

Sci. Adv. **9**, eadf3471 (2023)
DOI: 10.1126/sciadv.adf3471

The PDF file includes:

Figs. S1 to S7
Legends for movies S1 and S2

Other Supplementary Material for this manuscript includes the following:

Movies S1 and S2

Supplemental information titles and legends

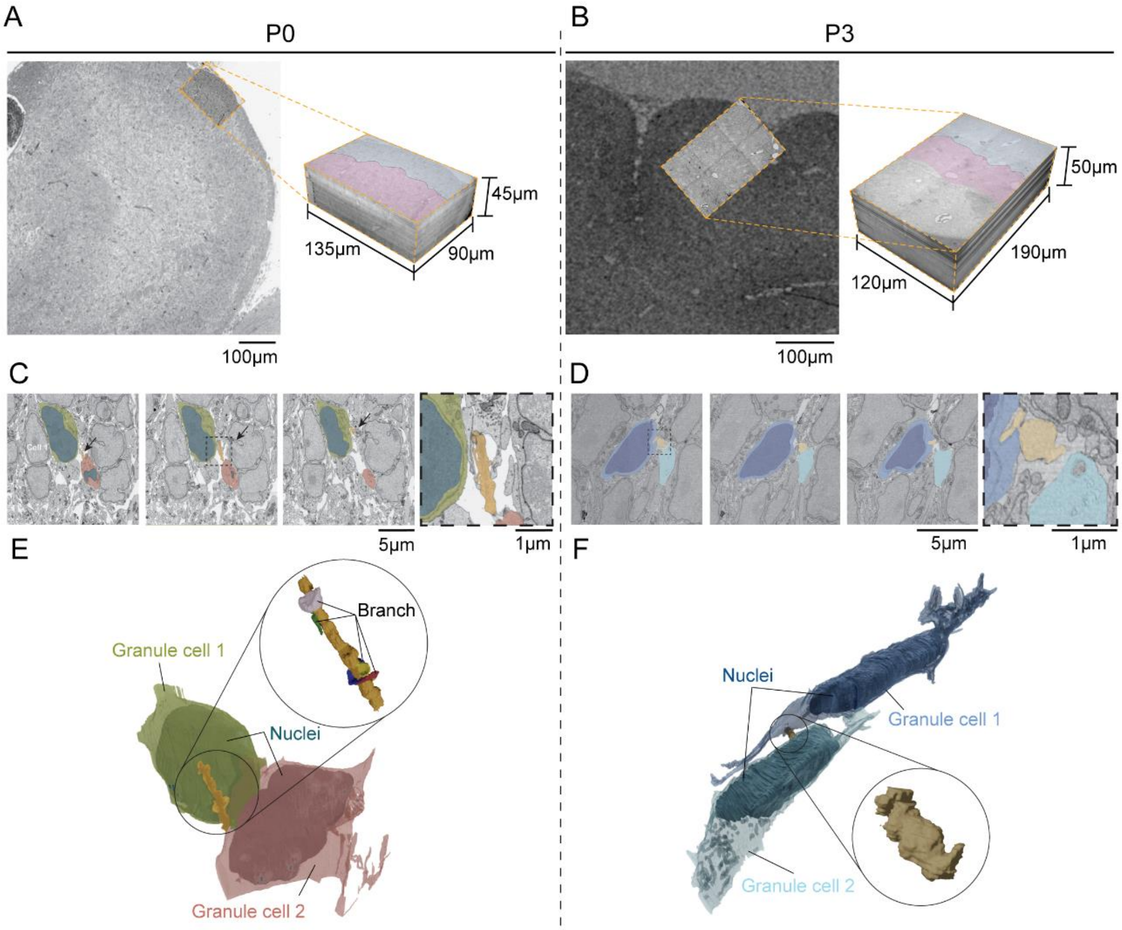

Fig. S1. Identification of cerebellar granule cell intercellular connectivity during post-natal development of mice

(**A**-**B**) Post-natal day 0 (A) and 3 (B) cerebellar cortex views obtained using serial sectioning scanning electron microscopy (ssSEM); electron micrographs (left) and 3D volume reconstructions (right). (**C**-**D**) Segmented electron micrographs of granule cells (GCs, various colors) and nuclei (blue) show intercellular connections (ICs, yellow, dashed box insets) bridging GCs in the external granule layer (EGL) of mice cerebella at P0 (C) and P3 (D). (**E**-**F**) 3D reconstruction of C and D, respectively, showing ICs connecting GCs; IC of E contains branches that emanate from the body of the IC.

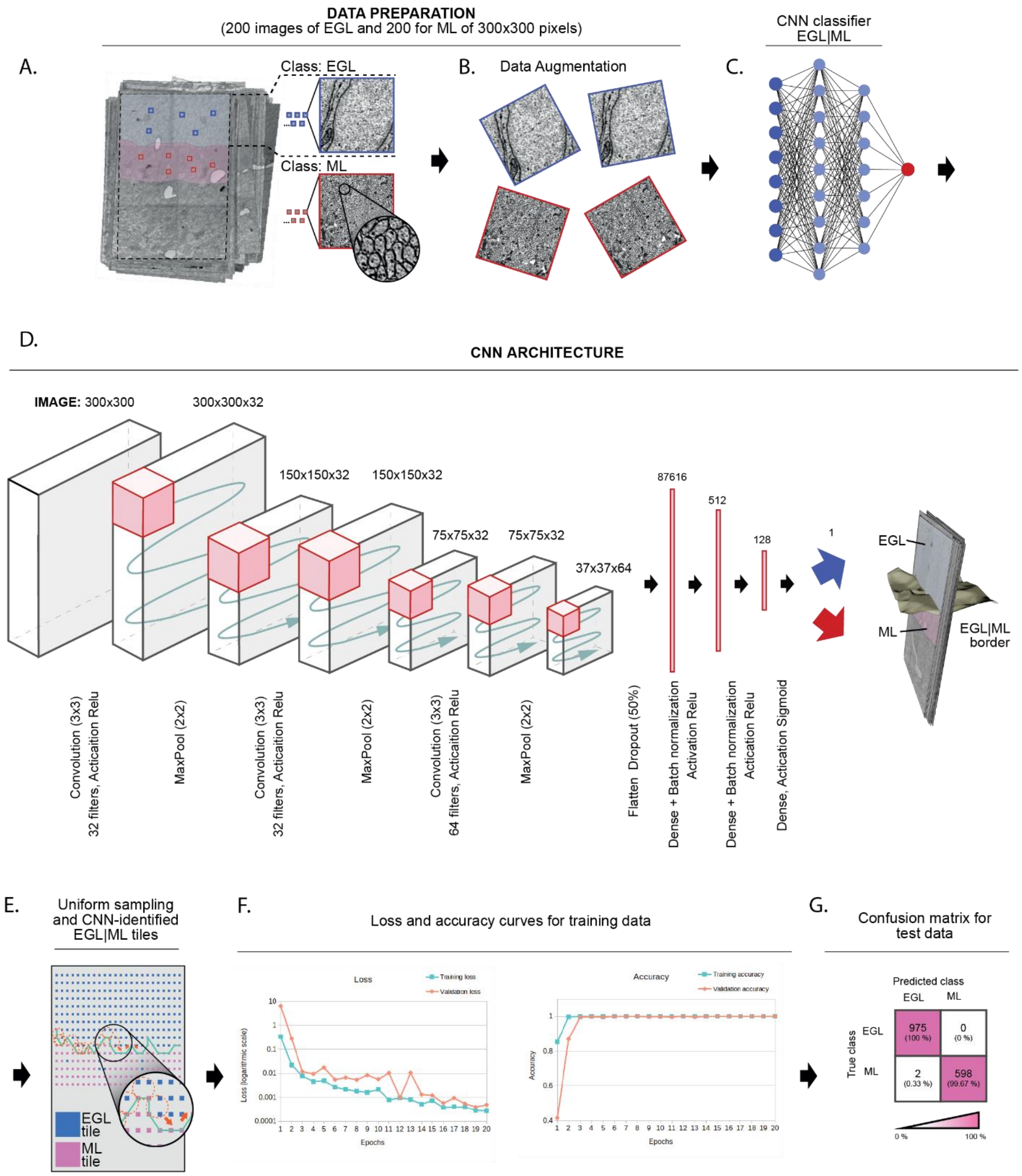
DATA PREPARATION
(200 images of EGL and 200 for ML of 300x300 pixels)
CNN classifier
EGL|ML
A.
Class: EGL
Class: ML
B.
Data Augmentation
C.
D.
CNN ARCHITECTURE
IMAGE: 300x300
300x300x32
150x150x32
150x150x32
75x75x32
75x75x32
37x37x64
87616
512
128
1
EGL
ML
EGL|ML border
Convolution (3x3) 32 filters, Acticaition Relu
MaxPool (2x2)
Convolution (3x3) 32 filters, Acticaition Relu
MaxPool (2x2)
Convolution (3x3) 64 filters, Acticaition Relu
MaxPool (2x2)
Flatten Dropout (50%)
Dense + Batch normalization Activation Relu
Dense + Batch normalization Actication Relu
Dense, Actication Sigmoid
E.
Uniform sampling and CNN-identified EGL|ML tiles
EGL tile
ML tile
F.
Loss and accuracy curves for training data
Loss
Training loss
Validation loss
Loss (logarithmic scale)
Epochs
Accuracy
Training accuracy
Validation accuracy
Epochs
G.
Confusion matrix for test data
Predicted class
EGL
ML
True class
EGL
ML
975 (100 %)
0 (0 %)
2 (0.33 %)
598 (99.67 %)
0 %
100 %

Fig. S2. Overview of the training process of the convolutional neural network (CNN) designed to identify EGL|ML boundary (see supplementary methods for details)

(A) Generation of training dataset: Manually chosen and labelled tiles for both EGL and ML classes from the ssSEM volume. **(B)** Data augmentation. **(C, D)** Architecture of the trained CNN classifier and the final result. **(E)** Predicted labels (EGL/ML) for uniformly sampled tiles allowing the segregation of the two layers. **(F)** Training performance of the CNN- Loss and Accuracy curves saturating at close to zero and one respectively indicating satisfactory training. **(G)** Testing performance of the CNN- Confusion matrix showing near-perfect identification of EGL and ML classes in the test dataset.

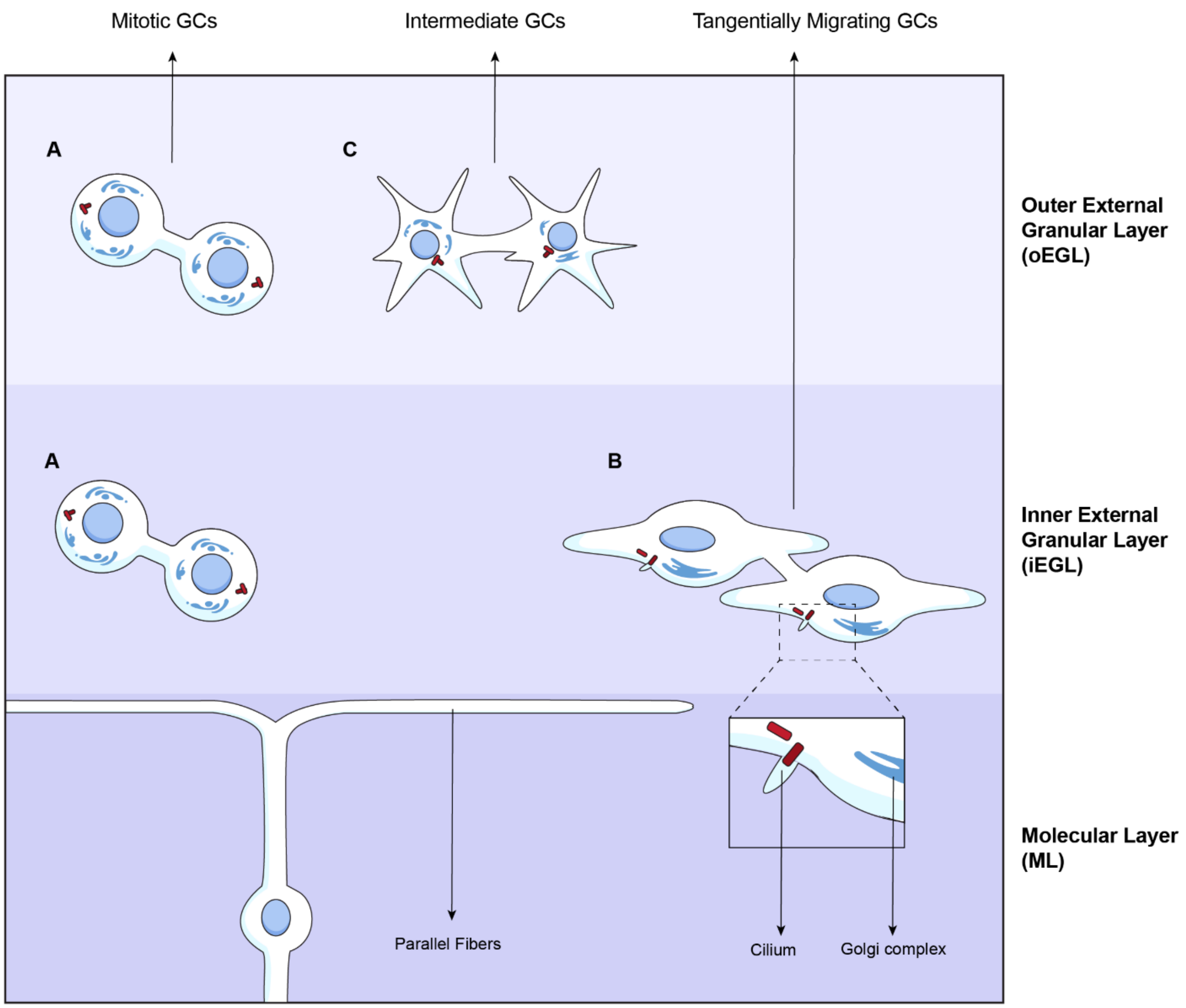

Fig. S3. Categorization of GCs into three categories based on organelles and morphology of cells

Connected GCs were grouped into mitotic, intermediate and migrating categories based on cell shape, number of Golgi complexes, presence of cilium, position of the centrosome in a cell and orientation of the protrusions of a cell when present (categorization provided in **Fig. 4E**). **(A)** Connected mitotic GCs were mainly present in the oEGL, but some are also localized in the iEGL. They display more than two Golgi complexes dispersed in the cell, absence of a cilium, largely spheroid shape and centrosomes positioned at opposite poles of the cells. **(B)** Connected tangentially migrating GCs are mainly localized in the iEGL. They harbor less than 2 Golgi complexes, have a cilium and show well-developed lamellipodia oriented parallel to the EGL|ML

boundary and parallel fibers. **(C)** Intermediate GCs were essentially observed in the oEGL. They share features of mitotic and migrating GCs. They show both filopodial and lamellipodial protrusions that are not specifically oriented in a particular direction, may have more than 2 Golgi complexes and a cilium; however, centrosomes are present in the soma, indicating non-migratory behavior.

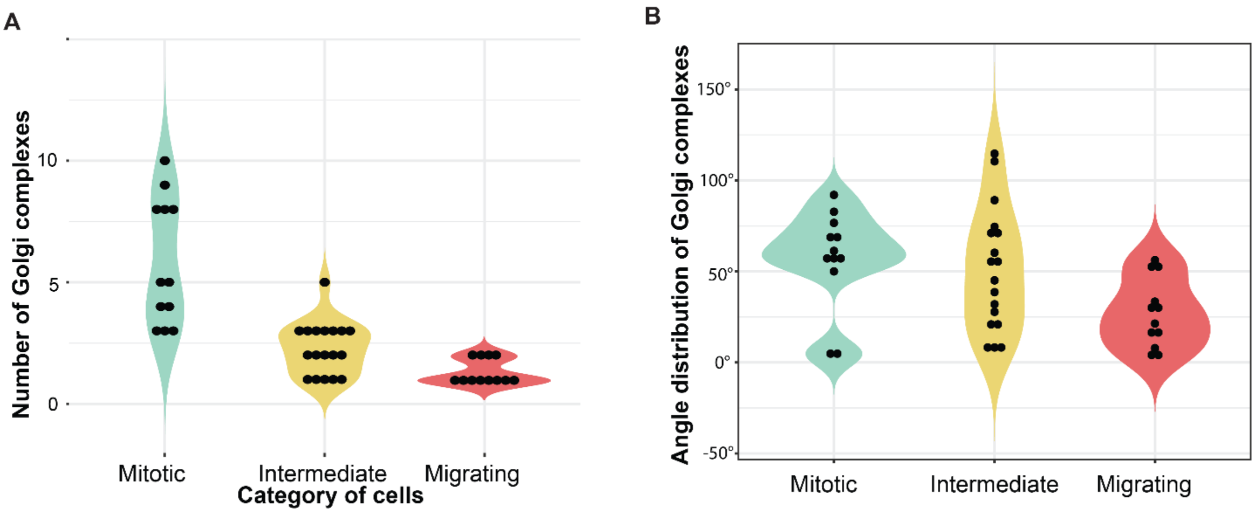

Fig. S4. Morphometric characterization of IC-connected GCs among the different categories

(A) Number of Golgi complexes observed in GCs of different categories (mitotic, intermediate, and migrating). In mitotic GCs, the Golgi complex is fragmented (3 to 10 individual Golgi complexes observed) compared to the Migrating GCs (1 to 2 individual Golgi complexes observed). **(B)** Angular distribution of Golgi complexes within GC. Angular distribution is calculated as the mean of the angles between the center of the nucleus of the GC and each voxel of the Golgi complexes in the cell. In mitotic GCs, Golgi complexes were homogeneously distributed in scattered clusters with most GCs displaying angular values above 50°, whereas most of the migrating GCs show angular values of Golgi complex distribution below 50° indicating well-formed Golgi complex.

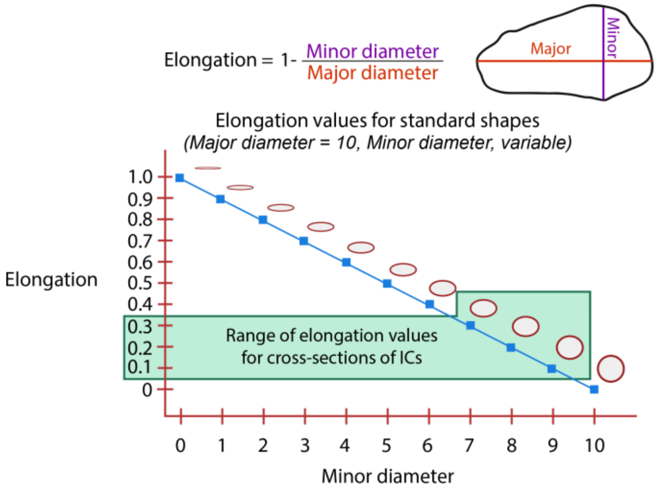

Fig. S5. Calculation of elongation of cross-sections

Elongation values of standard ellipses indicate that the observed cross-sections of our ICs were fairly circular, as shown by green highlighted region.

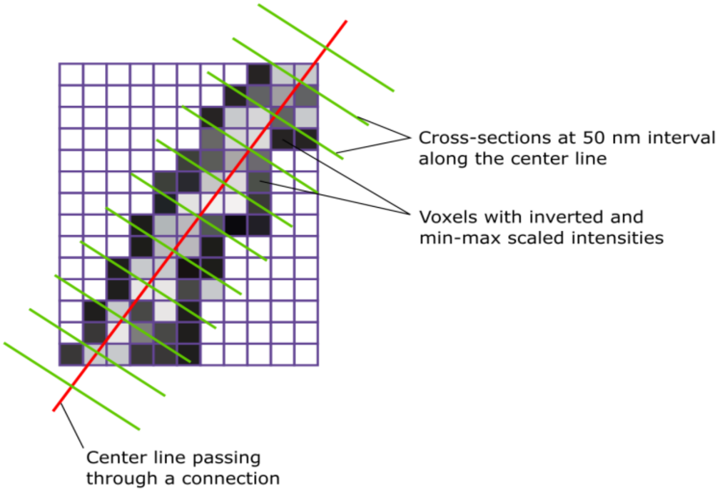

Fig. S6. Voxel intensity analysis within ICs

Schematic 2D representation of cross-sections (indicated by green lines) of an IC/connection (dark voxels) with respect to a center-line (red). The intensities of voxels are min-max scaled and averaged for each cross-section to obtain a voxel intensity profile along the IC that indicates the varying electron density along its length.

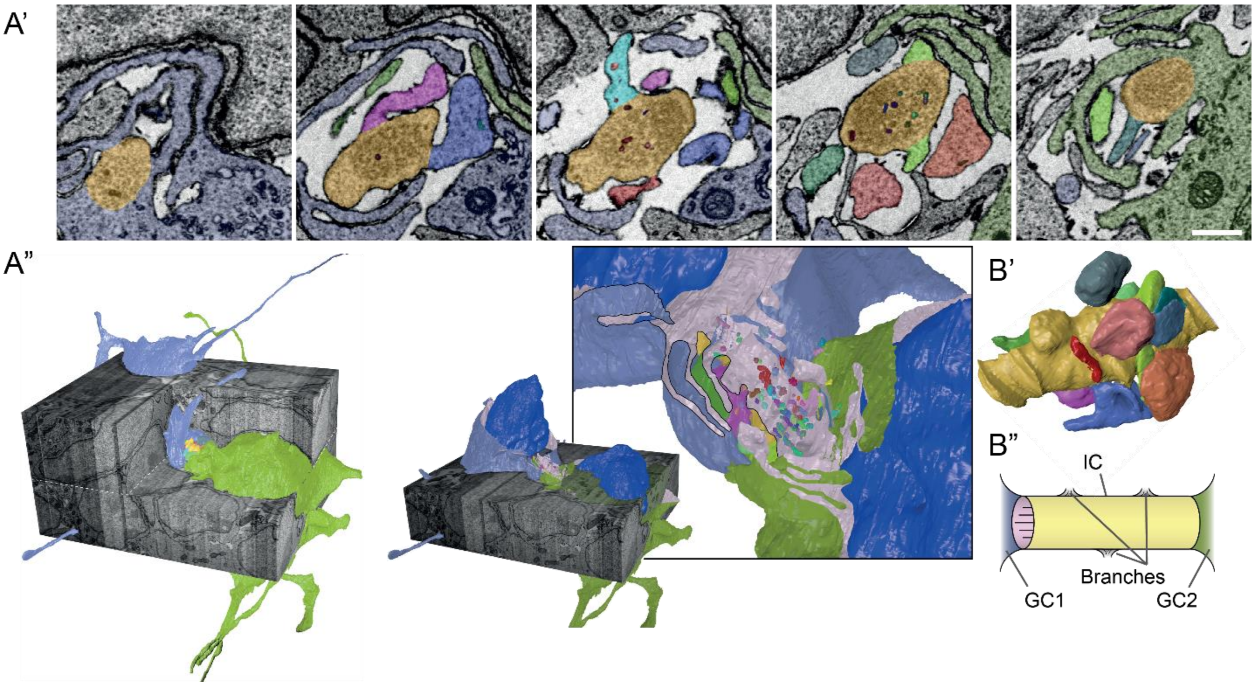

Fig. S7. Subcellular elements associated with ICs

(A) ssSEM micrographs **(A')** and 3D reconstructions **(A")** showing GCs (blue and green) connected by IC (yellow) that contains numerous cargoes (vesicles, red arrow; long membranous compartments, cyan arrow, **A"**), and extending branch protrusions containing cargoes (magenta arrow, **A'**). **(B)** 3D reconstruction **(B')** and diagram **(B")** of IC in A and its internal structure and contents, showing the branches that extend throughout.

Movie S1. 3D volume of a P7 mouse cerebellum imaged using ssSEM

Movie S2. GCs in EGL connected by ICs
